# Apoptosis in non-obstructive azoospermia: pathway crosstalk, cell-type vulnerability, and translational implications

**DOI:** 10.3389/fendo.2026.1683905

**Published:** 2026-06-26

**Authors:** Guoxiong Ma, Jialiang Deng, Xiaoqi Zhou, Weijin Chen, Nanhui Chen

**Affiliations:** Meizhou Hospital, Shantou University Medical College, Meizhou, Guangdong, China

**Keywords:** apoptosis, biomarkers, maturation arrest, non-obstructive azoospermia, oxidative stress, Sertoli cell-only syndrome, sperm retrieval, spermatogenesis

## Abstract

Non-obstructive azoospermia (NOA) is a severe form of male-factor infertility characterized by absent spermatozoa in the ejaculate due to impaired or absent spermatogenesis. Histologically, NOA includes Sertoli-cell-only syndrome, maturation arrest, and hypospermatogenesis, each reflecting distinct patterns of germ cell depletion and residual spermatogenic potential. Apoptosis is consistently observed in impaired spermatogenesis, but its biological meaning is context dependent. In physiological spermatogenesis, apoptosis maintains germ cell homeostasis and eliminates genetically or developmentally defective cells. In NOA, excessive or misdirected apoptotic signaling may contribute to germ cell loss, Sertoli cell dysfunction, Leydig cell impairment, and collapse of the testicular microenvironment. This narrative and critical review synthesizes current evidence on apoptotic regulation in NOA, focusing on intrinsic mitochondrial pathways, extrinsic death receptor signaling, checkpoint-mediated apoptosis, oxidative stress, and pathway crosstalk within the testicular niche. We evaluate cell-type-specific vulnerability in germ cells, Sertoli cells, and Leydig cells and examine upstream regulators including genetic abnormalities, hormonal dysregulation, oxidative stress, inflammation, epigenetic alterations, varicocele, heat stress, and environmental injury. We also assess apoptosis-related and apoptosis-adjacent biomarkers for predicting sperm retrieval during testicular sperm extraction and discuss therapeutic strategies aimed at correcting upstream stressors or restoring the spermatogenic microenvironment. Current evidence supports a multi-hit model in which genetic vulnerability, endocrine dysfunction, oxidative injury, epigenetic instability, inflammation, and somatic niche failure converge to determine whether apoptosis is protective, irreversible, or potentially reversible. Future progress will require validated biomarker panels, single-cell and spatial omics, and etiology-based patient stratification before apoptosis-targeted interventions can be safely translated into routine clinical care.

## 1 Introduction

Male infertility contributes substantially to the global burden of infertility and is implicated in approximately half of affected couples (1, 2). Among male-factor conditions, non-obstructive azoospermia (NOA) represents one of the most challenging entities because spermatozoa are absent from the ejaculate as a consequence of impaired or absent spermatogenesis rather than ductal obstruction (1, 2). Clinically, sperm retrieval by conventional testicular sperm extraction or microdissection testicular sperm extraction (micro-TESE), followed by intracytoplasmic sperm injection, remains the main route to biological fatherhood for many affected men (3–6). However, sperm retrieval is variable, and the current clinical parameters do not reliably identify all patients with focal residual spermatogenesis (3, 7–9).

The etiology of NOA is heterogeneous and includes chromosomal abnormalities, Y-chromosome microdeletions, monogenic defects affecting meiosis or germ cell differentiation, endocrine disorders, varicocele, cryptorchidism, infection, environmental exposure, heat stress, and gonadotoxic injury (2, 10–21). Nevertheless, a substantial proportion of cases remain idiopathic after routine clinical evaluation (10, 11). Histopathologically, NOA is usually classified into Sertoli-cell-only syndrome (SCOS), maturation arrest (MA), and hypospermatogenesis (HS). SCOS is characterized by seminiferous tubules containing Sertoli cells but lacking germ cells, MA refers to the initiation of spermatogenesis followed by arrest at a defined developmental stage, and HS indicates quantitatively reduced spermatogenesis with multiple germ cell stages still present (1, 2). These patterns are clinically relevant because they correlate, although imperfectly, with the probability of sperm retrieval (3, 7–9).

Apoptosis is a highly regulated form of programmed cell death that maintains tissue homeostasis and eliminates damaged or developmentally incompetent cells (22, 23). During normal spermatogenesis, apoptosis controls the germ cell number, maintains an appropriate germ-cell-to-Sertoli cell ratio, and removes germ cells with replication, recombination, chromosomal, or DNA damage defects (22, 24–26). Therefore, apoptosis in the testis is not inherently pathological. The central question in NOA is whether apoptotic signaling remains within physiological quality control or becomes excessive, persistent, or misdirected.

Human biopsy studies have linked impaired spermatogenesis to increased apoptotic indices, altered Bax/Bcl-2 ratios, Fas/Fas ligand activation, active caspase-3 expression, and stage-specific germ cell loss (25–29). These observations support a close association between apoptosis and spermatogenic failure, but they do not prove that apoptosis is always the primary cause of NOA. In genetic or meiotic forms of NOA, apoptosis may represent appropriate elimination of defective germ cells (10–15). In contrast, in cases associated with oxidative stress, varicocele, endocrine dysfunction, inflammation, heat stress, or environmental injury, apoptosis may include a potentially modifiable stress-induced component (20, 21, 30–37).

**Figure 1:**
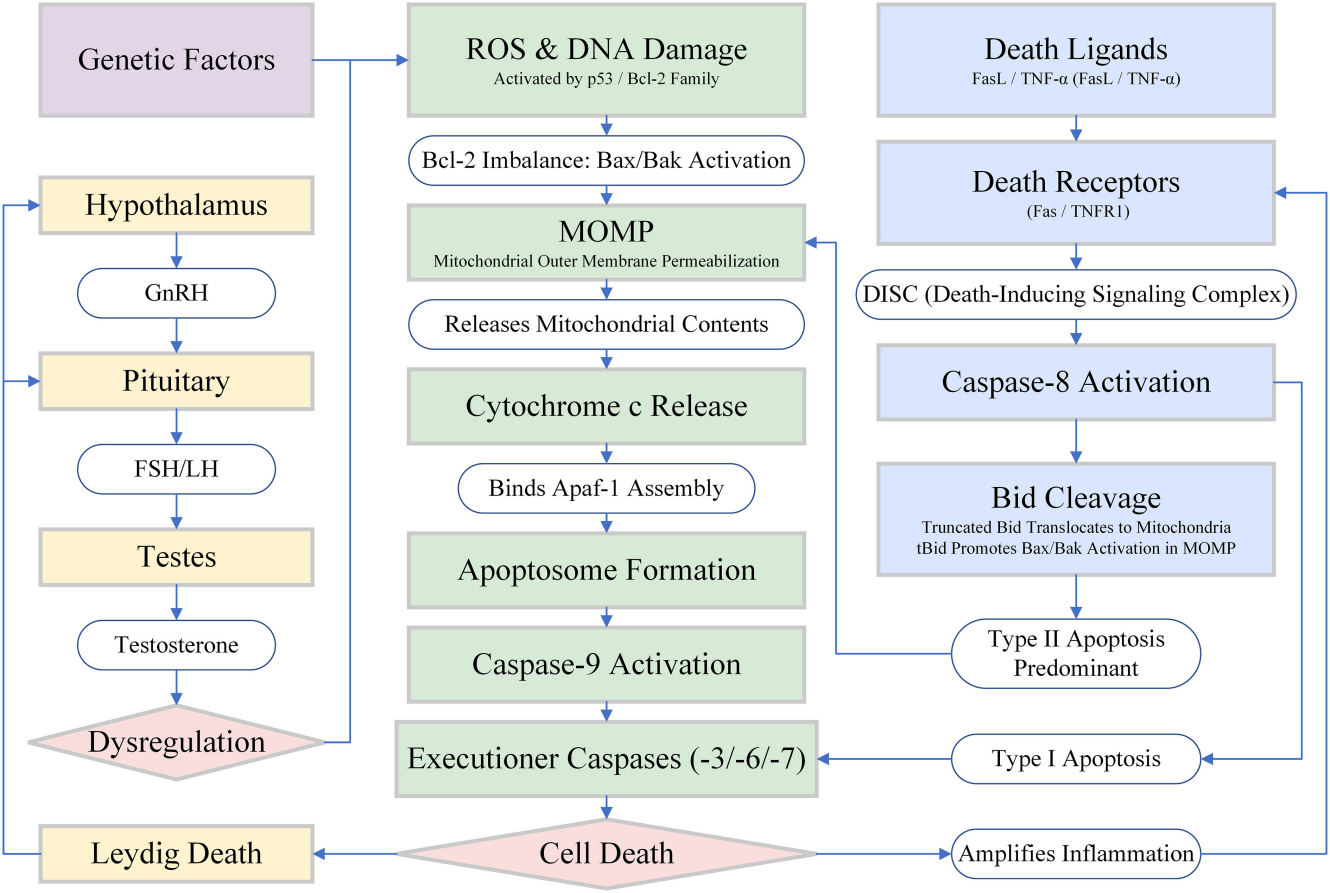
Integrated schematic of apoptotic pathway crosstalk in the NOA testis. This schematic summarizes the relationship among upstream genetic factors, hypothalamic–pituitary–gonadal axis dysregulation, oxidative stress, DNA damage, intrinsic mitochondrial apoptosis, extrinsic death receptor signaling, and downstream caspase-mediated cell death. Genetic vulnerability and endocrine dysregulation can increase reactive oxygen species production and DNA damage, thereby shifting the Bax/Bcl-2 family balance toward Bax/Bak activation, mitochondrial outer membrane permeabilization, cytochrome c release, apoptosome formation, caspase-9 activation, and executioner caspase activation. In parallel, death ligands such as FasL and TNF-α activate death receptors, the death-inducing signaling complex, and caspase-8. Caspase-8 may either directly activate executioner caspases or cleave Bid, thereby amplifying mitochondrial apoptosis. The figure also highlights Leydig cell injury and inflammatory amplification as feedback mechanisms that may worsen the testicular microenvironment.

This review approaches apoptosis in NOA as a convergent cellular response rather than a single disease pathway. We propose that genetic vulnerability, endocrine dysfunction, oxidative stress, epigenetic dysregulation, inflammation, environmental injury, and microenvironmental collapse interact to determine the apoptotic threshold of the seminiferous epithelium (10–21, 30–43). This distinction is clinically important because apoptosis may represent protective elimination of defective germ cells in some patients but potentially reversible stress-induced depletion in others. The molecular crosstalk among upstream triggers, intrinsic and extrinsic apoptotic pathways, hormonal signaling, and cellular outcomes is summarized in **Figure 1**.

## 2 Methods

This article is a narrative and critical review of the literature on apoptosis in non-obstructive azoospermia. Literature searches were performed in PubMed, Web of Science, and Google Scholar using combinations of the following terms: “non-obstructive azoospermia”, “NOA”, “apoptosis”, “germ cell apoptosis”, “Sertoli cell apoptosis”, “Leydig cell”, “Fas/Fas ligand”, “Bax”, “Bcl-2”, “caspase”, “oxidative stress”, “epigenetics”, “single-cell RNA sequencing”, “spatial transcriptomics”, “micro-TESE”, and “sperm retrieval biomarkers”. The final search was conducted on May 27, 2026.

Peer-reviewed English-language studies and authoritative reference chapters published or updated mainly between January 2000 and May 2026 were considered, with earlier foundational studies retained when they established key concepts in testicular apoptosis or spermatogenic failure. Human studies, systematic reviews, meta-analyses, authoritative reviews, and clinically relevant cohort studies were prioritized. Mechanistic, preclinical, or indirect evidence was included when it provided insight not available from human NOA studies; when such evidence was used, it was identified as preclinical, mechanistic, or indirect. Studies were excluded when they were not directly relevant to NOA, lacked sufficient methodological detail, or supported only speculative claims.

Because the available evidence is heterogeneous in the study design, patient selection, histological classification, molecular assays, and sperm retrieval outcome definitions, no formal meta-analysis or risk-of-bias assessment was performed. This review therefore does not claim to be a PRISMA-compliant systematic review. Evidence was instead synthesized narratively with attention to study type, biological plausibility, reproducibility across studies, and relevance to human NOA. Where evidence derives mainly from animal models, single-center cohorts, or indirect biomarker studies, this limitation is stated explicitly.

## 3 Molecular pathways of apoptosis in the NOA testis

Before examining how apoptosis contributes to NOA, it is necessary to define the main molecular systems through which programmed cell death is executed in the testis. Apoptosis is an energy-dependent process characterized by cell shrinkage, chromatin condensation, nuclear fragmentation, membrane blebbing, and non-inflammatory phagocytic clearance (23). Two major pathways—the intrinsic mitochondrial pathway and the extrinsic death receptor pathway—converge on executioner caspases that dismantle cellular structures (22, 23, 44–46). Additional systems, including p53-mediated checkpoint control, inhibitors of apoptosis proteins, oxidative stress signaling, and endoplasmic reticulum stress responses, modify the threshold for apoptotic commitment (33–36, 47–50). As illustrated in **Figure 1**, these pathways should be understood as an interconnected network rather than isolated linear cascades.

### 3.1 Intrinsic mitochondrial apoptosis

The intrinsic apoptotic pathway is initiated by intracellular stress signals such as DNA damage, oxidative injury, growth-factor withdrawal, heat stress, metabolic stress, hormonal deprivation, and meiotic checkpoint failure (22, 23, 44–46). In testicular germ cells, this pathway is particularly important because spermatogenesis involves repeated mitotic divisions, meiotic recombination, chromatin remodeling, and mitochondrial remodeling, all of which create opportunities for quality-control checkpoints (22, 24, 51).

The central regulatory step is mitochondrial outer membrane permeabilization (MOMP). This process is controlled by the balance between pro-apoptotic Bcl-2 family proteins, including Bax, Bak, Bad, Bim, PUMA, and Noxa, and anti-apoptotic members such as Bcl-2, Bcl-xL, and Mcl-1 (44–46). When cellular stress shifts this balance toward pro-apoptotic dominance, Bax and Bak oligomerize in the mitochondrial outer membrane, leading to the release of cytochrome c into the cytosol (44–46). Cytochrome c binds apoptotic protease-activating factor 1 to form the apoptosome, which activates caspase-9. Caspase-9 then activates executioner caspases, particularly caspase-3 and caspase-7, resulting in DNA fragmentation, cytoskeletal breakdown, and cellular dismantling (23, 44–46).

In NOA and other forms of impaired spermatogenesis, studies have reported an increased Bax/Bcl-2 ratio and activation of downstream caspases (25–29). These findings are most consistent in samples with MA and severe germ cell depletion. Nevertheless, Bax/Bcl-2 imbalance should be interpreted as a marker of apoptotic susceptibility rather than as a proof of a single causal pathway. Similar molecular changes may arise from diverse upstream insults, including genetic checkpoint activation, oxidative stress, hormonal deprivation, and heat-induced injury (20, 30–37, 52–54).

### 3.2 Extrinsic death receptor apoptosis

The extrinsic apoptotic pathway is initiated by extracellular ligands binding to death receptors on the cell surface. In the testis, the Fas/Fas ligand system and the TNF-α/TNFR1 axis are among the best-described death receptor systems (25, 26, 28, 29). Ligand binding promotes receptor trimerization and recruitment of adaptor proteins such as Fas-associated death domain protein, which then recruits and activates pro-caspase-8 within the death-inducing signaling complex (22, 44, 45). Activated caspase-8 can directly activate executioner caspases or cleave Bid to truncated Bid, thereby linking death receptor activation to mitochondrial amplification (44–46).

The Fas/Fas ligand system has physiological functions in testicular homeostasis. Sertoli cells can express Fas ligand and may participate in the elimination of excessive, damaged, or developmentally inappropriate germ cells (25, 26, 29). Under pathological conditions, however, this system may contribute to excessive germ cell apoptosis. Increased Fas/Fas ligand expression and active caspase-3 staining have been reported in human testes with MA and SCOS (28). These findings support a role for death receptor signaling in abnormal spermatogenesis but do not exclude the simultaneous activation of mitochondrial apoptosis (22, 25, 26, 28, 29).

Inflammatory cytokines may also engage extrinsic apoptotic pathways. TNF-α, IL-1β, and related cytokines generated by testicular macrophages, leukocytes, or chronically inflamed tissue can promote death receptor signaling and oxidative stress (21, 36). In such contexts, extrinsic apoptosis may function as a bridge between immune activation and germ cell depletion.

### 3.3 p53-mediated checkpoint apoptosis

The tumor suppressor p53 integrates DNA damage, replication stress, nucleotide imbalance, and meiotic defects into cell-cycle arrest or apoptosis (47, 48). In spermatogenesis, p53-mediated apoptosis is particularly relevant during meiotic prophase, when homologous recombination and synapsis must be completed accurately (14, 15, 47, 48). Cells with unresolved DNA breaks, asynapsis, or recombination failure may be eliminated through p53-regulated transcriptional programs involving Bax, PUMA, Noxa, and other apoptotic mediators (45–48).

This mechanism is especially relevant to MA. Genetic defects affecting meiotic recombination, synaptonemal complex formation, or DNA repair may trigger checkpoint-mediated apoptosis at the spermatocyte stage (10–15). Therefore, p53 activation in NOA should not always be interpreted as a pathological event that should be suppressed. In many genetic forms of maturation arrest, p53-mediated apoptosis may represent appropriate quality control.

### 3.4 Inhibitors of apoptosis proteins and survivin

Inhibitors of apoptosis proteins, including survivin, XIAP, and cIAPs, restrain caspase activation and help determine the threshold for apoptotic execution (49). Survivin is expressed in proliferating germ cells and has been implicated in the survival of spermatogonia and early spermatocytes (49). Reduced or altered survivin expression has been reported in impaired spermatogenesis, suggesting that loss of anti-apoptotic buffering may contribute to excessive cell death (49).

However, IAP-related findings remain less clinically developed than Bax/Bcl-2, Fas/Fas ligand, or caspase-3 data. Survivin and related proteins are best viewed as mechanistic contributors to apoptotic threshold rather than validated diagnostic or prognostic markers for routine clinical use.

### 3.5 Endoplasmic reticulum stress and stress-responsive modulators

Endoplasmic reticulum (ER) stress may contribute to testicular cell vulnerability, particularly in steroidogenically active Leydig cells and metabolically stressed Sertoli cells (33, 51, 55–57). Sustained activation of the unfolded protein response can induce CHOP-mediated repression of Bcl-2 signaling and promote mitochondrial apoptotic commitment (33–36). Oxidative protein damage, inflammatory stress, and steroidogenic overload may all increase ER stress.

Direct evidence that ER-stress markers are consistently elevated in human NOA Leydig cells remains limited. Therefore, ER stress should currently be presented as a biologically plausible and experimentally supported mechanism rather than as a validated human NOA-specific pathway. This distinction is important because overstatement of indirect or preclinical evidence may weaken the review’s credibility.

### 3.6 Pathway crosstalk and interpretation

The apoptotic machinery in the testis is characterized by extensive pathway crosstalk. Mitochondrial apoptosis appears to be the major convergence point for many NOA-relevant stressors, while death receptor signaling, p53-mediated checkpoint control, IAP regulation, oxidative stress, and ER stress modify apoptotic thresholds (22–29, 33–36, 44–50). The main limitation of the current literature is that most human studies examine isolated markers in cross-sectional biopsies (27, 28, 55, 57). Such designs identify associations but do not establish whether a specific pathway is primary, secondary, protective, or pathological in individual patients. The main apoptotic pathways discussed in this section are summarized in **Table 1**.

**Table 1:** Evidence-weighted summary of apoptotic pathways relevant to NOA.

Pathway	Main triggers	Key mediators	Testicular relevance	Interpretation	Key references
Intrinsic mitochondrial apoptosis	ROS, DNA damage, heat stress, growth-factor withdrawal, hormonal deprivation	Bax, Bak, Bcl-2, Bcl-xL, cytochrome c, Apaf-1, caspase-9, caspase-3/7	Major pathway for germ cell apoptosis and meiotic checkpoint-related elimination	Bax/Bcl-2 imbalance and caspase activation are recurrent findings in impaired spermatogenesis, but causality varies by etiology	(22–24, 27, 28, 44–46)
Extrinsic death receptor signaling	FasL, TNF-α, inflammatory cytokines	Fas, FasL, TNFR1, FADD, caspase-8, Bid/tBid	Regulates germ cell elimination and may link inflammation/Sertoli signaling to apoptosis	Fas/FasL and active caspase-3 are increased in MA and SCOS; best interpreted as contributory rather than exclusive	(21, 25, 26, 28, 29)
p53-mediated checkpoint apoptosis	DNA damage, meiotic asynapsis, replication stress	p53, PUMA, Noxa, Bax, p21	Meiotic surveillance, especially at spermatocyte stages	Highly relevant to MA and genetic defects affecting recombination or DNA repair	(14, 15, 47, 48)
IAP/survivin axis	Developmental stage and proliferative status	Survivin, XIAP, cIAPs, Smac/DIABLO	Anti-apoptotic threshold in germ cells	Survivin expression is associated with human germ cells and apoptosis control; not a stand-alone diagnostic marker	(49)
ER-stress/UPR-associated apoptosis	Proteotoxic stress, oxidative injury, steroidogenic stress	PERK, IRE1α, ATF6, CHOP, Bcl-2 suppression	Plausible contributor to Leydig/Sertoli cell vulnerability	Plausible mechanism supported mainly by mechanistic, preclinical, or indirect evidence	(33–36, 51)
Pathway crosstalk	Death receptor activation in type II cells	Caspase-8, Bid/tBid, Bax/Bak	Amplifies death receptor signals through mitochondria	Supports the mitochondrial pathway as a convergence point for diverse upstream insults	26, 29, 44–46]

NOA, non-obstructive azoospermia; ROS, reactive oxygen species; MA, maturation arrest; SCOS, Sertoli-cell-only syndrome; UPR, unfolded protein response; IAP, inhibitor of apoptosis protein; ER, endoplasmic reticulum; FADD, Fas-associated death domain protein.

## 4 Cell-type-specific vulnerability and histological phenotypes

The seminiferous epithelium is a multicellular system in which germ cells, Sertoli cells, Leydig cells, peritubular myoid cells, immune cells, and vascular elements interact to support spermatogenesis (51). Apoptosis affecting one compartment may compromise the stability of the others. For this reason, NOA should not be understood only as germ cell loss; it is often accompanied by the progressive disruption of the somatic niche and interstitial endocrine support (51, 55–57).

When interpreting cell-type-specific markers in NOA, it is important to distinguish apoptosis markers from functional or compartmental markers. TUNEL staining, active caspase-3, Bax/Bcl-2 imbalance, p53 activation, and DNA fragmentation are more directly related to apoptotic signaling. By contrast, PLZF and ID4 mainly indicate the spermatogonial stem/progenitor compartment; connexin-43, inhibin B, and vimentin reflect Sertoli cell organization or function; and StAR, 3β-HSD, and testosterone/LH-related indices reflect Leydig cell steroidogenic status. These markers are useful for defining cellular vulnerability and microenvironmental collapse, but they should not be interpreted as apoptosis-specific markers unless accompanied by direct evidence of apoptotic execution. This distinction is emphasized in **Table 2** to avoid conflating cell identity, functional deterioration, and programmed cell death.

**Table 2:** Cell-type-specific apoptosis and evidence hierarchy in NOA.

Cell type	Main apoptotic/stress triggers	Dominant pathway or process	Representative/associated markers	Evidence level and caution	Functional consequence	NOA subtype relevance	Key references
Spermatogonia	Genetic defects, niche-factor withdrawal, oxidative stress, epigenetic disruption	Intrinsic and p53-associated stress responses	p53↑, Bax↑, caspase-3↑; PLZF/ID4 as spermatogonial compartment markers	Direct apoptosis markers are stronger than PLZF/ID4. PLZF/ID4 changes indicate stem/progenitor compartment alteration rather than apoptosis specifically	Reduced regenerative reserve; progressive germ cell depletion	Partial SCOS, early MA, severe HS	(27, 28, 43, 47, 48, 60)
Primary spermatocytes	Meiotic recombination failure, chromosomal asynapsis, DNA damage	p53-mediated and mitochondrial apoptosis	Bax/Bcl-2↑, γH2AX↑, active caspase-3↑	Strong biological plausibility and good fit with MA, but human studies remain mostly cross-sectional	Meiotic arrest and failure to generate haploid cells	MA, especially spermatocyte-stage arrest	(14, 15, 24, 27, 28, 47, 48)
Spermatids	Testosterone withdrawal, chromatin remodeling defects, oxidative stress	Hormone-sensitive mitochondrial apoptosis	Caspase-3↑, DNA fragmentation; protamine imbalance as indirect spermiogenic marker	Protamine changes are not apoptosis-specific and should be interpreted as spermiogenic dysfunction	Incomplete spermiogenesis; reduced focal sperm production	Late MA, HS	(24, 30, 31, 51)
Sertoli cells	Germ cell loss, oxidative stress, inflammatory cytokines, androgen/FSH signaling impairment	Intrinsic and cytokine-associated apoptosis; functional deterioration	Active caspase-3↑, Fas/FasL changes, vimentin disorganization, connexin-43↓, inhibin B↓	Connexin-43 and inhibin B indicate Sertoli dysfunction rather than apoptosis per se	BTB disruption, loss of paracrine support, impaired immune privilege	SCOS > MA > HS	(21, 28–31, 51)
Leydig cells	Inflammation, interstitial fibrosis, oxidative stress from steroidogenesis, LH dysregulation	Mitochondrial stress, inflammatory signaling; ER stress plausible	StAR↓, 3β-HSD↓, testosterone/LH ratio↓, lipofuscin↑, structural degeneration	Human NOA evidence supports altered Leydig morphology/function more strongly than direct apoptosis quantification	Reduced intratesticular testosterone; secondary impairment of Sertoli and germ cell survival	Variable across subtypes; often progressive	(30, 31, 51, 55–57)

NOA, non-obstructive azoospermia; SCOS, Sertoli-cell-only syndrome; MA, maturation arrest; HS, hypospermatogenesis; BTB, blood–testis barrier; FSH, follicle-stimulating hormone; LH, luteinizing hormone; ER, endoplasmic reticulum.

### 4.1 Germ cell vulnerability across histological phenotypes

Germ cells are the primary cellular target of apoptotic dysregulation in NOA (22, 24, 27, 28). During normal spermatogenesis, apoptosis eliminates a substantial proportion of germ cells to preserve genomic integrity and maintain an appropriate relationship between germ cells and Sertoli cell support capacity (22, 24, 51). In NOA, this physiological process may become excessive or may occur in response to irreversible developmental defects (10–15, 27, 28).

In MA, apoptosis is often concentrated at or near the stage of arrest (27, 28). Primary spermatocytes are particularly vulnerable because meiotic recombination and chromosome synapsis impose stringent quality-control requirements (14, 15, 47). Apoptotic elimination at this stage may reflect unresolved DNA damage, chromosomal asynapsis, or recombination failure. This pattern is consistent with the frequent association between MA and genetic defects involving meiotic regulators (10–15).

In SCOS, interpretation is more difficult because most or all germ cells are absent by the time biopsy is performed. In partial SCOS, residual spermatogonia or focal germ cell populations may show apoptotic features, suggesting that progressive depletion of the stem/progenitor compartment may contribute to the establishment of the SCOS phenotype (27, 28). This has clinical relevance because focal residual spermatogenesis may still permit sperm retrieval in selected patients (3, 7–9).

In HS, apoptosis may be increased across multiple stages, but germ cells remain present throughout the spermatogenic lineage (27). This pattern suggests quantitative reduction rather than complete lineage interruption and is generally associated with a better probability of sperm retrieval than complete SCOS or complete early MA (3, 7–9).

A central unresolved question is whether germ cell apoptosis in NOA is primary or secondary. In genetic forms such as complete AZFa or AZFb deletions, severe meiotic defects, or DNA repair disorders, apoptosis may eliminate cells that cannot produce functional spermatozoa (10–15). In contrast, in NOA associated with varicocele, oxidative stress, inflammation, or endocrine imbalance, at least part of the apoptotic burden may be stress-induced and potentially modifiable (20, 21, 30–37). This distinction should guide the interpretation of biomarkers and the design of therapeutic studies.

### 4.2 Sertoli cell dysfunction and niche collapse

Sertoli cells provide structural support, metabolic substrates, paracrine factors, androgen-responsive signaling, and blood–testis barrier integrity (51). They are essential for spermatogonial maintenance, meiotic progression, spermiogenesis, and immune privilege (51). Although adult Sertoli cells are relatively resistant to apoptosis, Sertoli cell dysfunction and occasional apoptosis have been described in impaired spermatogenesis (28, 29, 51, 55–57).

In NOA, Sertoli cell injury may occur through several mechanisms. Loss of germ cell contact can destabilize Sertoli cell function, a concept sometimes referred to as the “empty niche” effect. Oxidative stress and inflammatory cytokines may directly damage Sertoli cells or disrupt tight junctions (20, 21, 33–37). Deficient androgen or FSH signaling may reduce survival and metabolic support (30, 31). Altered expression of connexin-43, inhibin B, vimentin organization, and other Sertoli-associated markers may indicate functional impairment, although these markers are not apoptosis-specific (28, 30, 31, 51).

The consequences of Sertoli cell dysfunction are broader than the loss of a support cell population. Blood–testis barrier disruption can expose meiotic and post-meiotic germ cells to immune recognition (21, 51). Reduced paracrine support may impair spermatogonial self-renewal and differentiation (43, 51). Decreased inhibin B secretion reflects loss of Sertoli function and contributes to altered hypothalamic–pituitary–gonadal feedback (30, 31). Thus, Sertoli cell injury may amplify initial germ cell damage and convert focal spermatogenic failure into a more generalized testicular disorder.

### 4.3 Leydig cell dysfunction and endocrine vulnerability

Leydig cells support spermatogenesis by producing testosterone under luteinizing hormone stimulation (51, 55–57). Intratesticular testosterone concentrations are substantially higher than serum concentrations and are required for meiotic progression, spermiogenesis, Sertoli cell function, and blood–testis barrier maintenance (30, 31, 51). Leydig cell dysfunction can therefore worsen germ cell and Sertoli cell vulnerability even when Leydig cells are not the primary site of disease.

In NOA, Leydig cell changes include altered morphology, clustering, hyperplasia-like patterns, lipofuscin accumulation, impaired steroidogenic enzyme expression, interstitial remodeling, and altered endocrine output (55–57). Some studies suggest apoptotic or degenerative Leydig cell changes in abnormal spermatogenesis, but the strength of direct human evidence varies (55–57). It is therefore more accurate to describe Leydig cell involvement as structural and functional impairment with possible apoptotic contribution rather than as uniform Leydig cell apoptosis in all NOA subtypes.

Mechanistically, Leydig cells may be vulnerable to oxidative stress generated during steroidogenesis, inflammatory cytokines from macrophages, chronic LH stimulation, and ER stress related to high secretory demand (21, 33, 51, 55–57). When Leydig cell function declines, reduced intratesticular testosterone can secondarily impair Sertoli cell support and germ cell survival, creating a feed-forward loop (30, 31, 51).

### 4.4 Histology-linked cellular cascade

The cell-type-specific patterns described above support a cellular cascade model of NOA progression. In this model, primary germ cell apoptosis may be initiated by genetic, meiotic, oxidative, endocrine, epigenetic, or environmental stressors (10–21, 30–43). Germ cell depletion then weakens Sertoli cell–germ cell communication, disrupts Sertoli cell function, and compromises the blood–testis barrier (28, 29, 51). Sertoli dysfunction alters paracrine support and endocrine feedback, while Leydig cell impairment reduces intratesticular testosterone and further weakens spermatogenesis (30, 31, 51, 55–57). Over time, these interactions may create an irreversible loop of germ cell loss, somatic niche failure, and endocrine insufficiency.

This model is clinically useful because it emphasizes timing and reversibility. Interventions are most likely to succeed when residual germ cells and functional support cells remain. Once complete germ cell depletion, severe tubular hyalinization, and irreversible microenvironmental collapse have occurred, anti-apoptotic therapy is unlikely to restore spermatogenesis (3–9). The cell-type-specific apoptotic patterns and their functional implications are summarized in **Table 2**.

## 5 Etiology-specific triggers and the multi-hit model

**Figure 2:**
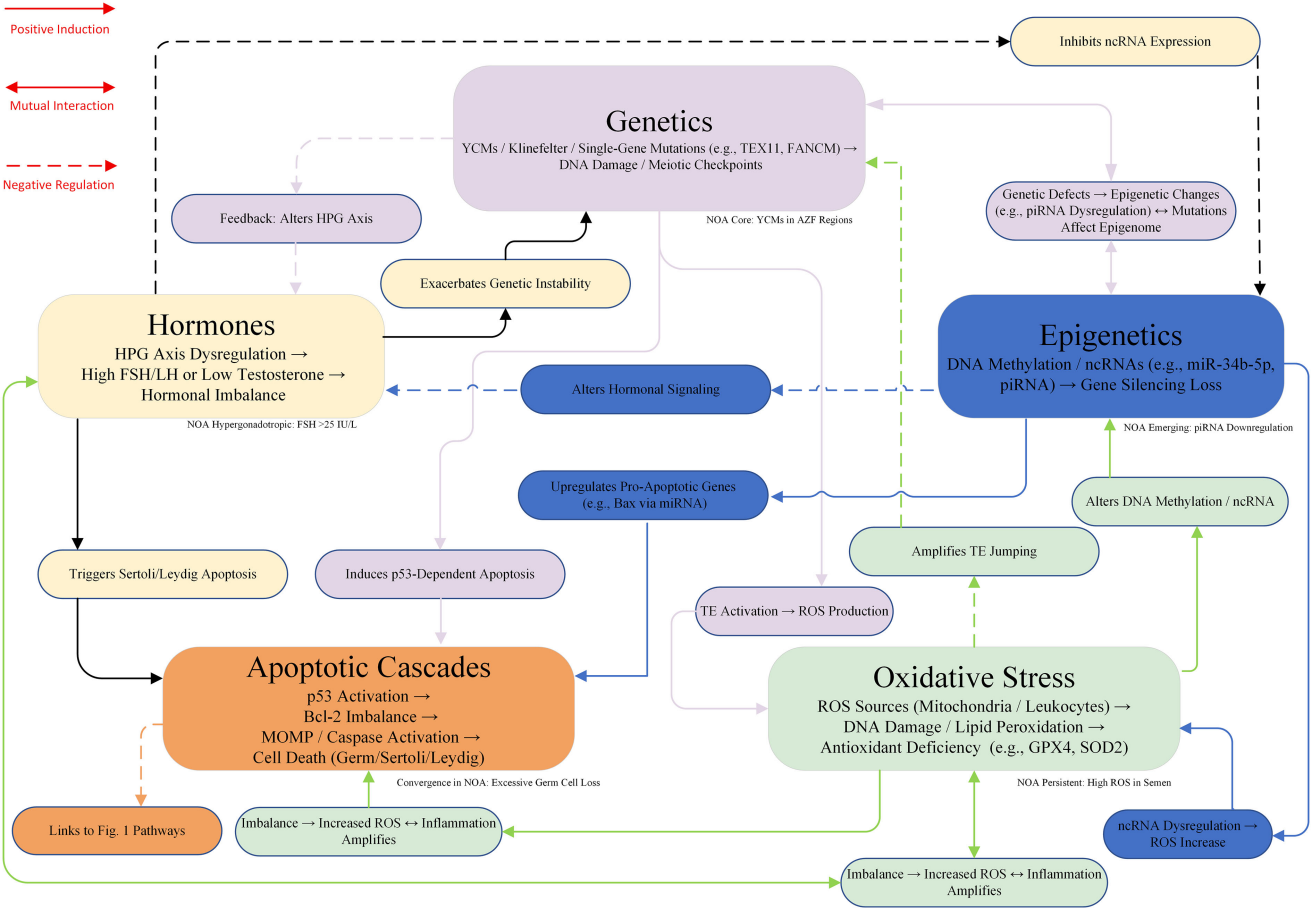
Multi-hit network linking upstream regulators to apoptotic cascades in NOA. This network diagram illustrates how genetic defects, hormonal dysregulation, oxidative stress, and epigenetic alterations interact to promote apoptotic activation in the NOA testis. Genetic abnormalities may predispose germ cells to DNA damage, meiotic checkpoint activation, and epigenetic instability. Hormonal imbalance may impair Sertoli and Leydig cell support and alter the hypothalamic–pituitary–gonadal axis feedback. Oxidative stress can induce DNA damage, lipid peroxidation, mitochondrial dysfunction, and inflammatory amplification. Epigenetic dysregulation, including altered DNA methylation, non-coding RNAs, and piRNA-related transposon control, may further modify apoptotic thresholds. These processes together converge on p53 activation, Bax/Bcl-2 imbalance, mitochondrial outer membrane permeabilization, caspase activation, and cell death. Solid arrows indicate positive induction, bidirectional arrows indicate mutual interaction, and dashed arrows indicate inhibitory or negative regulatory relationships.

Apoptosis in NOA is activated by diverse upstream regulators. These regulators rarely operate in isolation; they instead interact to determine the apoptotic threshold of germ cells and somatic support cells. The major categories include genetic defects, hormonal dysregulation, oxidative stress, inflammation, environmental and physical stressors, and epigenetic alterations (10–21, 30–43). **Figure 2** presents this multi-hit network, emphasizing reciprocal interactions among genetic susceptibility, endocrine imbalance, oxidative injury, epigenetic instability, and apoptotic cascades.

### 5.1 Genetic factors

Genetic abnormalities represent one of the best-established etiological categories in NOA. They include chromosomal aneuploidies, Y-chromosome microdeletions, copy-number changes, and monogenic defects affecting meiosis, DNA repair, germ cell differentiation, or Sertoli–germ cell communication (10–19).

Y-chromosome azoospermia factor deletions are clinically important because they carry direct diagnostic and prognostic implications (10–13). Complete AZFa deletions typically result in severe germ cell depletion or SCOS histology and are associated with extremely poor sperm retrieval prognosis. Complete AZFb deletions are commonly associated with maturation arrest and poor sperm retrieval. AZFc deletions show more variable phenotypes, ranging from severe oligozoospermia to NOA, and sperm retrieval may be possible in selected patients (10–13). The severity gradient reflects the developmental stage at which essential germ cell functions are disrupted.

Klinefelter syndrome is a common chromosomal cause of NOA (16, 17). Germ cell loss in Klinefelter syndrome is progressive and may begin before or during puberty. Mechanisms include sex chromosome dosage effects, impaired meiotic sex chromosome pairing, Sertoli cell dysfunction, Leydig cell impairment, and progressive tubular degeneration (16, 17). The presence of focal residual spermatogenesis explains why micro-TESE may retrieve sperm in selected patients, although outcomes vary according to age, testicular volume, endocrine profile, surgical technique, and center experience (16, 17).

Next-generation sequencing has identified the monogenic causes of NOA involving genes such as TEX11, SYCP3, and HSF2 and DNA repair-related pathways (14, 15, 18, 19). TEX11 variants can impair meiotic crossover formation and activate pachytene checkpoint apoptosis (14, 15). Synaptonemal complex defects may trigger asynapsis-related germ cell elimination. DNA repair defects may lower the threshold for p53-mediated apoptosis. These findings support the concept that many cases previously labeled idiopathic may have an underlying genetic susceptibility (10, 11, 14, 15).

Genetic factors illustrate why apoptosis is not always a therapeutic target. In some genetic forms of NOA, apoptosis may represent appropriate quality control that prevents defective germ cells from progressing. However, variable phenotypes among patients with similar genetic lesions indicate that genetic predisposition interacts with modifier genes, epigenetic context, hormonal milieu, and environmental exposures (10–15, 38–43). The genetic contributors and their implications for apoptosis and sperm retrieval are summarized in **Table 3**.

**Table 3:** Genetic factors in NOA and implications for apoptosis and sperm retrieval.

Genetic factor	Approximate relevance in NOA	Apoptosis-related mechanism	Typical histology	TESE prognosis	Evidence and caution	Key references
Complete AZFa deletion	Rare among NOA; clinically important	Loss of genes required for early germ cell survival and spermatogonial maintenance	Usually complete SCOS	Very poor; sperm retrieval generally not expected	Exact SRR estimates are cohort-dependent; complete AZFa deletion is generally associated with extremely poor sperm retrieval prognosis	(10–13)
Complete AZFb deletion	Rare among NOA; clinically important	Meiotic arrest through impaired germ cell RNA processing and spermatocyte checkpoint activation	Spermatocyte-stage MA	Very poor; sperm retrieval generally not expected in complete AZFb deletion	Complete AZFb deletions should be distinguished from partial or combined deletion patterns	(10–13)
AZFc deletion	Most common AZF deletion	Defective germ-cell transcript regulation; variable residual spermatogenesis	MA or HS; sometimes focal spermatogenesis	Variable; often possible in selected patients	SRR varies widely by cohort, surgical technique, histology, and deletion pattern	(3–6, 10–13)
Klinefelter syndrome, 47,XXY	Common chromosomal cause of NOA	X-dosage effects, sex chromosome pairing defects, progressive germ cell loss, Leydig dysfunction	Progressive hyalinization/SCOS with focal spermatogenesis	Variable; approximately 20–50% in selected cohorts	Age, testicular volume, endocrine profile, center experience, and surgical technique influence SRR	(16, 17)
TEX11 variants	Uncommon but important in meiotic arrest	Defective meiotic crossover formation; pachytene checkpoint activation	MA, often pachytene	Poor to uncertain	Evidence is gene- and variant-specific	(14, 15)
SYCP3 and synaptonemal-complex gene variants	Rare	Asynapsis and meiotic checkpoint-mediated apoptosis	Zygotene/pachytene MA	Poor to uncertain	Evidence is limited by small family-based or cohort-based studies	(10, 11, 19)
DNA repair/Fanconi pathway variants	Rare but increasingly detected by sequencing	Lowered threshold for DNA damage-induced apoptosis	Variable	Unknown or variable	Emerging genetic category; not yet an established prognostic marker	(10, 11, 19)
Antioxidant defense polymorphisms	Variable association	Reduced ROS buffering and increased oxidative susceptibility	Variable	Not established	Susceptibility evidence; not currently a validated TESE predictor	(37)

SRR ranges vary substantially across studies because of differences in histological sampling, micro-TESE expertise, patient age, testicular volume, endocrine status, and genetic subtype definition. Exact percentages should be retained only when directly supported by the cited study.

NOA, non-obstructive azoospermia; AZF, azoospermia factor; SCOS, Sertoli-cell-only syndrome; MA, maturation arrest; HS, hypospermatogenesis; TESE, testicular sperm extraction; SRR, sperm retrieval rate.

### 5.2 Hormonal dysregulation

Spermatogenesis requires coordinated hypothalamic–pituitary–gonadal axis signaling. FSH supports Sertoli cell function and spermatogonial maintenance, while LH stimulates Leydig cell testosterone production (30, 31, 51). Intratesticular testosterone is essential for meiotic progression, spermiogenesis, and Sertoli cell function (30, 31, 51).

In NOA, elevated serum FSH is common and usually reflects impaired Sertoli cell function and reduced inhibin B feedback (7, 8, 30–32). Although elevated FSH can indicate severe spermatogenic failure, it does not exclude focal spermatogenesis or the possibility of sperm retrieval (8). Similarly, serum testosterone provides only an indirect estimate of the intratesticular androgen environment. Some patients with NOA may have correctable endocrine abnormalities, including low testosterone, abnormal testosterone-to-estradiol ratio, or hypogonadotropic components (30–32, 58).

Hormonal deprivation can promote germ cell apoptosis (24, 30, 31). Testosterone withdrawal particularly affects spermatocytes and spermatids, while inadequate FSH support may impair Sertoli cell survival signaling and paracrine support (30, 31, 51). Hormonal optimization with clomiphene citrate, aromatase inhibitors, hCG, or FSH has been used empirically before sperm retrieval in selected patients (30–32, 58). Existing studies suggest possible benefit in defined subgroups, but evidence remains heterogeneous and patient selection is crucial. Hormonal therapy should not be presented as a universal anti-apoptotic treatment for NOA.

### 5.3 Oxidative stress

Oxidative stress is one of the most widely studied contributors to male infertility and testicular apoptosis (33–36). The testis is susceptible to oxidative injury because germ cells proliferate rapidly, sperm membranes contain abundant polyunsaturated fatty acids, mitochondrial activity is high, and steroidogenesis generates reactive oxygen species (33–36). When reactive oxygen species (ROS) production exceeds antioxidant defenses, oxidative damage affects lipids, proteins, DNA, and mitochondria.

ROS can activate apoptosis through several routes. Oxidative damage to mitochondrial membranes promotes MOMP (33–36, 44–46). Cardiolipin oxidation facilitates cytochrome c release. DNA oxidation activates p53-dependent apoptotic programs (47, 48). ROS can also activate stress kinases such as JNK and p38 MAPK and amplify inflammatory signaling (20, 21, 33–37). In germ cells, these mechanisms may converge on Bax upregulation, Bcl-2 suppression, caspase activation, and DNA fragmentation (22, 27, 28, 33–36).

Human studies have reported elevated oxidative stress markers and reduced antioxidant capacity in male infertility and in subsets of NOA (33–37). However, oxidative stress should be interpreted as one component of a multifactorial process. Many studies are cross-sectional and cannot determine whether oxidative stress is a primary cause, secondary consequence, or both. Antioxidant trials have produced mixed results, likely because patients are not routinely stratified by oxidative stress burden, residual germ cell reserve, or histological subtype (68).

### 5.4 Environmental and physical stressors

Environmental and physical stressors may contribute to apoptosis in susceptible testes. Varicocele is a common and potentially correctable condition associated with scrotal hyperthermia, venous stasis, hypoxia, accumulation of metabolites, and oxidative stress (20, 52). In selected azoospermic or severely oligozoospermic patients, varicocele repair may improve the testicular microenvironment, although outcomes are variable and not all patients benefit (20).

Heat stress is a well-established trigger of germ cell apoptosis (53, 54, 59). The testis normally functions below core body temperature, and elevated temperature can activate intrinsic mitochondrial pathways, p53 signaling, and Fas/Fas ligand-related mechanisms (53, 54, 59). Pachytene spermatocytes are particularly heat-sensitive. Clinically relevant sources include cryptorchidism, varicocele, febrile illness, occupational heat exposure, and lifestyle factors that increase scrotal temperature (20, 53, 54).

Toxins, endocrine disruptors, heavy metals, smoking, obesity-related inflammation, and medications may also increase oxidative and apoptotic stress (20, 21, 33–37). The strength of evidence varies by exposure, dose, duration, and patient susceptibility. In idiopathic NOA, environmental exposures may act as second hits in genetically or epigenetically vulnerable individuals.

### 5.5 Inflammation and immune-mediated injury

Inflammation can promote apoptosis through cytokine signaling, oxidative stress, and blood–testis barrier disruption (21, 36). TNF-α, IL-1β, IL-6, and other inflammatory mediators may activate death receptor signaling or sensitize germ cells to mitochondrial apoptosis (21, 25, 26, 28, 29). Activated leukocytes and macrophages generate ROS, creating synergy between inflammatory and oxidative pathways (21, 36).

Chronic orchitis, subclinical inflammation, infection, and immune dysregulation may therefore contribute to impaired spermatogenesis in some patients (21). However, the proportion of NOA cases attributable to inflammation remains uncertain because inflammatory changes may be focal, transient, or difficult to detect in routine evaluation. More standardized assessment of testicular immune signatures is needed.

### 5.6 Epigenetic alterations

Epigenetic mechanisms regulate germ cell differentiation, meiotic progression, chromatin remodeling, and transposon silencing (38–43). DNA methylation, histone modifications, piRNA pathways, miRNAs, lncRNAs, circRNAs, and other non-coding RNAs are increasingly implicated in spermatogenic failure (38–43). In the context of **Figure 2**, epigenetic alterations should be interpreted not only as independent upstream triggers but also as amplifiers of oxidative stress, inflammatory signaling, and genetic vulnerability.

Aberrant DNA methylation can disrupt imprinted genes, repetitive element silencing, and germ cell survival programs (38, 39). Defects in piRNA-mediated transposon repression may increase genomic instability and apoptotic susceptibility (39, 40). miRNAs can modulate apoptotic thresholds by targeting genes involved in calcium signaling, mitochondrial regulation, and stress responses (42, 67).

Recent single-cell and multi-omics studies have begun to identify cell-type-specific transcriptional and epigenetic abnormalities in NOA. These approaches reveal heterogeneity that cannot be captured by bulk tissue analysis and suggest that residual germ cell competence must be interpreted within its somatic niche context (43, 60)—for example, integrated RNA-seq, single-cell RNA-seq, single-cell chromatin accessibility, and spatial transcriptomic analyses have identified dysfunctional Wnt signaling in spermatogonia from NOA samples (60). These findings support the view that apoptosis may arise not only from intrinsic germ cell defects but also from disturbed niche signaling and impaired developmental competence.

Epigenetic abnormalities may be both causes and consequences of apoptosis. Primary epigenetic defects may disrupt germ cell development and trigger apoptosis, whereas cellular stress may induce secondary epigenetic changes that reinforce cell death. Distinguishing these possibilities requires longitudinal, spatial, or interventional approaches that remain technically challenging in human testicular tissue.

### 5.7 The multi-hit model

The upstream triggers of apoptosis in NOA are interconnected. Genetic defects may lower the threshold for oxidative stress (10–15). Endocrine insufficiency may weaken Sertoli cell support and sensitize germ cells to environmental injury (30–32). Oxidative stress may induce epigenetic instability and inflammatory signaling (20, 30–38). Epigenetic defects may amplify the impact of genetic or hormonal perturbations (38–43). This supports a multi-hit model in which NOA emerges when cumulative stress exceeds the resilience of the spermatogenic system.

Several limitations should be emphasized. Most human studies are cross-sectional and cannot determine temporal sequence. Many studies examine one trigger or marker at a time, whereas clinical NOA likely involves interacting networks. Histological heterogeneity within the same testis complicates interpretation. Publication bias may overrepresent positive associations. Future research should therefore integrate genomic, epigenomic, transcriptomic, proteomic, metabolomic, endocrine, and histological data from the same patients (43, 60–62).

### 5.8 Integrative synthesis: apoptosis as a context-dependent threshold response

Taken together, the available evidence suggests that apoptosis in NOA should be interpreted as a context-dependent threshold response rather than a uniform pathogenic mechanism. Genetic defects, endocrine insufficiency, oxidative stress, inflammation, epigenetic instability, and somatic niche disruption can each lower the resilience of the spermatogenic system, but their clinical consequences depend on timing, cellular reserve, and histological context. In severe genetic or meiotic defects, apoptosis may function mainly as protective quality control by eliminating cells that cannot complete normal differentiation. In contrast, in patients with residual germ cells and modifiable stressors such as oxidative injury, endocrine imbalance, varicocele, or inflammation, apoptosis may include a potentially reversible component.

This framework helps reconcile apparently conflicting findings across studies: the same apoptotic marker may indicate irreversible developmental failure in one patient group but stress-induced and potentially modifiable damage in another. Therefore, future research should move beyond measuring apoptosis alone and should integrate apoptotic markers with genetic diagnosis, histological pattern, endocrine profile, oxidative stress status, and single-cell or spatial evidence of residual germ cell competence. This integrative interpretation also provides the rationale for etiology-oriented patient stratification, biomarker panel development, and cautious evaluation of apoptosis-targeted interventions.

## 6 Biomarkers and sperm retrieval prediction

Micro-TESE is the standard approach for attempting sperm retrieval in many men with NOA (3–6). Although histology, testicular volume, FSH, inhibin B, testosterone, genetic diagnosis, and age provide useful information, none can reliably predict sperm retrieval in all patients (3–9). Focal spermatogenesis may exist even in testes with severe overall damage. Understanding apoptosis has potential value for sperm retrieval prediction, but clinical application remains limited by heterogeneity, lack of standardized biomarkers, and uncertainty about whether apoptosis is reversible or protective in individual patients (3–9).

Tissue-based apoptotic markers, including TUNEL apoptotic index, Bax/Bcl-2 ratio, and active caspase-3 staining, are associated with spermatogenic impairment (27–29). A lower apoptotic burden may suggest preserved germ cell potential, whereas extensive apoptosis may indicate more severe damage. However, tissue-based markers require biopsy, are affected by spatial heterogeneity, and lack standardized thresholds. Their value is therefore greater for mechanistic understanding than routine preoperative prediction.

Serum markers such as FSH, inhibin B, LH, testosterone, and the testosterone-to-estradiol ratio indirectly reflect Sertoli and Leydig cell function (7, 8, 30–32). They are clinically accessible but insufficiently specific. High FSH often indicates severe spermatogenic failure but does not exclude focal sperm production (8). Inhibin B may reflect Sertoli cell function but varies by assay and clinical context. Testosterone may be influenced by obesity, sampling time, age, and systemic health (30–32, 58).

Seminal plasma and extracellular vesicle biomarkers offer a non-invasive window into the testicular and excurrent duct environment (7, 61–67). Candidate markers include cell-free DNA, cell-free RNA, exosomal miRNAs, tRNA-derived fragments, and germ cell-associated proteins (7, 61–67). These markers are promising but remain early-stage. Many studies involve small cohorts, variable normalization methods, and limited external validation (7, 61, 62, 65–67). Moreover, not all seminal liquid biopsy markers are apoptosis-specific; some reflect residual spermatogenic activity, accessory gland contribution, inflammation, or tissue remodeling (7, 62–64).

Combined models integrating clinical, hormonal, genetic, histological, and molecular data generally perform better than single markers (3, 6, 7). However, precise AUC values should only be reported when tied to specific studies. At present, combined biomarker panels are promising but not sufficiently standardized for routine clinical decision-making (7). The apoptosis-related and apoptosis-adjacent biomarkers are summarized in **Table 4**.

**Table 4:** Apoptosis-related and apoptosis-adjacent biomarkers for predicting sperm retrieval.

Biomarker category	Marker	Source	Relationship to sperm retrieval	Main limitations	Key references
Tissue-based apoptosis	TUNEL apoptotic index	Testicular biopsy	Higher apoptotic burden generally associates with poorer spermatogenic reserve	Invasive; spatial heterogeneity; not suitable as routine preoperative test	(27–29)
Tissue-based apoptosis	Bax/Bcl-2 ratio	Testicular biopsy	Higher pro-apoptotic ratio suggests more severe damage	Assay thresholds not standardized; small cohorts	(27–29)
Tissue-based apoptosis	Active caspase-3	Testicular biopsy	Increased staining indicates active apoptotic execution	Same limitations as TUNEL; not independently validated	(28, 29)
Serum endocrine markers	FSH, inhibin B, testosterone, LH/testosterone ratio	Blood	Reflect Sertoli/Leydig function and testicular reserve; predictive value is inconsistent	Poor specificity; thresholds vary; cannot reliably identify focal spermatogenesis alone	(7, 8, 30–32)
Seminal liquid biopsy	Cell-free DNA/cell-free nucleic acids	Seminal plasma	May reflect abortive spermatogenesis, apoptosis, necrosis, or inflammation	Pre-analytical variation; unclear tissue origin; limited external validation	(7, 61)
Seminal extracellular vesicles	Exosomal or EV-associated miRNAs/small RNAs	Seminal plasma	Candidate markers may reflect germ cell or somatic-cell status	Small cohorts; normalization issues; inconsistent panels	(65–67)
Seminal proteins	TEX101, ECM1, LDHC, PGK2, other germ cell proteins	Seminal plasma	Reflect presence or absence of germ cell/spermatogenic activity	More closely related to spermatogenic activity than to apoptosis specifically	(7, 62–64)
Combined models	Clinical + hormonal + genetic + histological ± molecular markers	multiple	Generally outperform single markers	AUC estimates are model- and cohort-dependent and require external validation	(3–9)

Combined models generally outperform single markers, but thresholds, external validation, and clinical utility remain insufficient for routine use.

NOA, non-obstructive azoospermia; TESE, testicular sperm extraction; FSH, follicle-stimulating hormone; LH, luteinizing hormone; EV, extracellular vesicle.

## 7 Therapeutic implications and safety concerns

No intervention has yet been validated as a broadly effective anti-apoptotic therapy for unselected NOA patients. Therefore, therapeutic strategies should be framed as etiology-directed correction of upstream stressors or empirical optimization of the testicular microenvironment rather than as proven universal anti-apoptotic treatment.

Antioxidant therapy is widely used because oxidative stress can activate mitochondrial apoptosis (33–36). Agents such as vitamin E, coenzyme Q10, N-acetylcysteine, L-carnitine, zinc, and other antioxidants may reduce oxidative burden in selected patients. However, evidence for improving sperm retrieval in NOA is inconsistent (68). Benefit is most plausible in patients with residual germ cells and measurable oxidative stress burden rather than in complete germ cell aplasia or irreversible genetic arrest.

Hormonal optimization aims to improve the endocrine environment supporting spermatogenesis. Clomiphene citrate, aromatase inhibitors, hCG, and FSH have been used in selected men, particularly those with low testosterone, abnormal testosterone-to-estradiol ratio, or hypogonadotropic features (30–32, 58). These approaches may reduce stress-induced apoptosis indirectly by restoring Sertoli and Leydig cell support. They should be framed as etiology-directed or empirical optimization, not as proven direct anti-apoptotic therapy for NOA.

Varicocele repair may reduce heat stress, venous stasis, hypoxia, and oxidative injury in selected patients (20, 52). Its role in NOA remains heterogeneous, but it is relevant as an upstream intervention that may reduce apoptosis by correcting a modifiable stressor. Patient selection is essential.

Direct anti-apoptotic drugs, such as caspase inhibitors or Bax/Bcl-2 modulators, remain preclinical (59). Although they can protect germ cells in experimental models, they raise important safety concerns. Apoptosis eliminates genetically damaged or developmentally incompetent germ cells; indiscriminate suppression could theoretically preserve abnormal cells (22–24, 47). In the reproductive context, this concern is amplified because the retrieved sperm may be used for intracytoplasmic sperm injection. For this reason, direct anti-apoptotic therapy should be considered experimental until patient selection, germline safety, and delivery specificity are addressed.

Growth factors, spermatogonial stem cell support, nanoparticle delivery, hydrogels, extracellular vesicle-based delivery, spermatogonial stem cell transplantation, *in vitro* spermatogenesis, and gene editing represent future strategies (43, 60, 69, 70). These approaches are scientifically important but remain distant from routine clinical NOA treatment. They are best interpreted as future perspectives rather than established therapies. The therapeutic strategies are grouped by translational maturity in **Table 5**.

**Table 5:** Therapeutic strategies grouped by translational maturity.

Category	Strategy	Mechanistic rationale	Evidence level	Clinical interpretation	Main challenges	Key references
Clinically used/empirical	Antioxidants, including vitamin E, CoQ10, NAC, L-carnitine, zinc	Reduce oxidative stress and mitochondrial injury	Clinical trials and reviews; heterogeneous quality	May benefit selected patients with oxidative stress burden, but evidence for improving SRR in NOA remains inconsistent	Dose, duration, patient selection, endpoint heterogeneity	(33–37, 68)
Clinically used/empirical	Hormonal optimization with clomiphene citrate, aromatase inhibitors, hCG ± FSH	Improve testosterone/estradiol balance, intratesticular testosterone, and Sertoli support	Retrospective studies, small trials, reviews	Potentially useful in selected patients with correctable endocrine abnormalities; not a universal NOA therapy	Variable response; limited evidence in primary testicular failure	(30–32, 58)
Etiology-directed supportive	Varicocele repair where clinically indicated	Reduces heat stress, venous stasis, and oxidative injury	Clinical evidence in selected NOA/azoospermic men	May improve the testicular microenvironment in selected patients, but response is heterogeneous	Selection criteria and spontaneous sperm appearance remain unpredictable	(20, 52)
Preclinical anti-apoptotic	Caspase inhibition	Blocks execution phase of apoptosis	Animal/*in vitro* studies	Mechanistically informative but not clinically translatable at present	Systemic toxicity; risk of preserving damaged germ cells; no human NOA data	(59)
Preclinical mitochondrial protection	Bax/Bcl-2 pathway modulation	Prevents MOMP and cytochrome c release	Mostly non-testicular or experimental evidence	Conceptually relevant but not established as a testis-specific therapy	Testis delivery; specificity; genetic safety	(44–46, 59)
Preclinical trophic support	GDNF, SCF, and other niche factors	Supports spermatogonial survival/self-renewal	Animal and *in vitro* systems	Promising for spermatogonial stem cell biology, but not ready for clinical NOA therapy	Local delivery, dosage, oncogenic or dysplastic risk	(43, 51, 60)
Future delivery platform	Nanocarriers, EVs, hydrogels, local delivery systems	Increase testicular bioavailability and limit systemic exposure	Preclinical development	Potential enabling technology rather than a proven NOA treatment	BTB penetration, biocompatibility, reproductive toxicity, regulatory path	(69, 70)
Future regenerative	Spermatogonial stem cell transplantation/*in vitro* spermatogenesis	Replaces or expands depleted germline reserve	Rodent proof-of-concept; early human translational work	Future strategy for selected patients with preserved or recoverable spermatogonial stem cells	Human spermatogonial stem cell culture, genetic/epigenetic safety, clinical regulation	(43, 60)
Future genetic correction	Gene editing for monogenic NOA	Corrects causal mutations	Theoretical/proof-of-concept	Scientifically plausible but ethically and technically distant from clinical use	Germline ethics, off-target effects, delivery to spermatogonial stem cells	(10, 11, 14, 15)

Strategies are grouped according to translational maturity rather than expected efficacy.

NOA, non-obstructive azoospermia; SRR, sperm retrieval rate; NAC, N-acetylcysteine; hCG, human chorionic gonadotropin; FSH, follicle-stimulating hormone; MOMP, mitochondrial outer membrane permeabilization; BTB, blood–testis barrier; EV, extracellular vesicle.

The central translational and safety challenge is identifying which patients have reversible stress-induced apoptosis and which have irreversible genetic or developmental failure. A patient with residual germ cells, oxidative stress, endocrine imbalance, or varicocele may plausibly benefit from upstream correction (20, 30–37, 58, 68). A patient with complete AZFa deletion, complete AZFb deletion, or severe meiotic gene disruption is unlikely to benefit from anti-apoptotic treatment because apoptosis may reflect appropriate quality control (10–15). Future clinical studies should therefore stratify patients by genetic diagnosis, histology, endocrine profile, oxidative stress burden, inflammatory status, and molecular evidence of residual germ cell competence (7, 43, 60). An etiology-oriented clinical stratification framework for interpreting apoptosis in NOA is proposed in **Table 6**.

**Table 6:** Etiology-oriented clinical stratification framework for interpreting apoptosis in NOA.

Clinical/etiological stratum	Dominant interpretation of apoptosis	Suggested evaluation	Translational implication	Counseling and sperm retrieval implication	Key caution
Complete AZFa deletion, complete AZFb deletion, or severe meiotic gene defects	Predominantly protective quality-control apoptosis or irreversible developmental elimination	Karyotype, Y-chromosome microdeletion testing, targeted gene panels or exome sequencing when indicated	Direct anti-apoptotic treatment is biologically inappropriate because apoptosis may eliminate defective germ cells	Very poor sperm retrieval expectation in complete AZFa/AZFb deletion; counseling should emphasize low likelihood of biological sperm retrieval	Do not interpret high apoptosis as a reversible therapeutic target in irreversible genetic arrest
AZFc deletion or Klinefelter syndrome with possible focal spermatogenesis	Mixed pattern: genetic vulnerability with focal residual germ cell survival	Genetic diagnosis, endocrine profile, testicular volume, ultrasound, micro-TESE planning	Etiology-specific counseling; endocrine optimization may be considered only when clinically indicated	Micro-TESE may retrieve sperm in selected patients, but outcome depends on histology, age, testicular reserve, and surgical expertise	Avoid applying uniform sperm retrieval estimates across genetically heterogeneous groups
Hypospermatogenesis or late maturation arrest with endocrine imbalance	Potentially modifiable stress-induced apoptosis superimposed on residual spermatogenesis	FSH, LH, total testosterone, estradiol, inhibin B, testosterone-to-estradiol ratio, clinical assessment for hypogonadotropic components	Hormonal optimization may improve the testicular microenvironment in selected patients	Residual germ cell reserve may justify micro-TESE after appropriate endocrine evaluation or optimization	Hormonal therapy should not be presented as a universal anti-apoptotic treatment for NOA
Varicocele-associated NOA or NOA with heat/oxidative stress burden	Potentially reversible oxidative or heat-induced apoptotic stress when residual germ cells remain	Physical examination, scrotal ultrasound, oxidative stress assessment when available, exposure history	Correction of upstream stressors, such as clinically significant varicocele, may reduce apoptotic pressure	Benefit is most plausible in patients with residual spermatogenesis rather than complete germ cell aplasia	Varicocele repair and antioxidants have heterogeneous outcomes and require careful patient selection
Inflammatory, infectious, or immune-associated NOA	Cytokine- and ROS-amplified death receptor and mitochondrial apoptosis	History of orchitis or infection, leukocytospermia when applicable, inflammatory markers, clinical evaluation for autoimmune or infectious causes	Treat identifiable infection or inflammation; avoid nonspecific immunomodulation without evidence	Potentially modifiable in selected cases, but the proportion of NOA directly attributable to inflammation remains uncertain	Inflammatory changes may be focal or secondary rather than causal
Idiopathic NOA with molecular evidence of residual germ cell competence	Apoptosis may be mixed: stress-induced, compensatory, or secondary to unidentified genetic/epigenetic defects	Expanded genetic testing, endocrine profile, oxidative stress assessment, seminal plasma or EV biomarkers, and, where available, single-cell/spatial signatures	Best suited for biomarker-panel development and mechanism-based stratification	Micro-TESE may be reasonable when clinical and molecular features suggest focal residual spermatogenesis	No single biomarker currently distinguishes protective from pathological apoptosis
Complete SCOS, severe tubular hyalinization, or extensive microenvironmental collapse	Apoptosis is likely a late marker of irreversible depletion or niche failure	Histology, testicular volume, endocrine reserve, prior biopsy or surgical findings if available	Anti-apoptotic strategies are unlikely to restore spermatogenesis once the germline niche has collapsed	Sperm retrieval probability is low, although focal spermatogenesis may still exist in selected heterogeneous testes	A single biopsy may miss focal sperm production; interpretation should account for spatial heterogeneity

NOA, non-obstructive azoospermia; SCOS, Sertoli-cell-only syndrome; FSH, follicle-stimulating hormone; LH, luteinizing hormone; EV, extracellular vesicle; ROS, reactive oxygen species; micro-TESE, microdissection testicular sperm extraction.

## 8 Future directions

The next stage of NOA research should move from descriptive marker cataloguing toward mechanism-based patient stratification. Priority areas include multicenter validation of preoperative biomarker panels, integration of genetic testing with endocrine and oxidative stress profiling, spatial and single-cell omics to identify residual competent germ cell niches, and prospective studies assessing whether the modification of upstream triggers changes apoptotic burden or sperm retrieval outcomes (7, 43, 60).

Several methodological limitations must be addressed. First, most human studies are cross-sectional biopsy studies and cannot define the temporal sequence from upstream insult to apoptotic activation and cellular depletion (27, 28, 55, 57). Second, testicular pathology in NOA is spatially heterogeneous, so a single biopsy may not represent the entire testis (3–9). Third, many studies analyze one pathway or marker at a time, whereas NOA is likely driven by interacting networks (10–21, 30–43). Fourth, animal models of cryptorchidism, heat stress, toxic injury, or genetic disruption provide mechanistic insight but do not fully reproduce the heterogeneity of human NOA (53, 54, 59). Finally, biomarker studies frequently suffer from small sample size, single-center design, non-standardized assay thresholds, and inconsistent definitions of sperm retrieval success (7–9, 61–67).

A key unresolved issue is whether apoptosis in a given patient is protective, pathological, or both. Physiological germ cell apoptosis is essential for maintaining genomic integrity, removing defective germ cells, and matching germ cell numbers to Sertoli cell support capacity (22, 24, 51). In genetic forms of NOA, such as severe AZF deletions or meiotic recombination defects, apoptosis may represent the appropriate elimination of cells that cannot complete normal differentiation (10–15). In this context, indiscriminate anti-apoptotic therapy would be biologically questionable and could theoretically preserve genetically damaged germ cells. By contrast, apoptosis associated with potentially modifiable stressors—such as oxidative stress, varicocele, inflammation, or endocrine imbalance—may include a reversible component (20, 21, 30–37).

At present, no validated biomarker can reliably distinguish protective quality-control apoptosis from pathological, stress-induced apoptosis in individual NOA patients (7). This represents a major translational bottleneck. Future biomarker panels should therefore not merely quantify apoptosis but should integrate apoptotic markers with genetic diagnosis, histological subtype, endocrine status, oxidative stress measures, and single-cell or spatial signatures of residual germ cell competence (7, 43, 60). Anti-apoptotic therapy should be pursued only after patient groups with reversible, stress-induced apoptosis can be distinguished from those in whom apoptosis reflects irreversible genetic or meiotic failure (10–15, 58, 59, 68–70).

## 9 Conclusion

Apoptotic dysregulation is a recurrent feature of NOA and may contribute to germ cell depletion, Sertoli cell dysfunction, and secondary impairment of Leydig cell support. However, apoptosis should not be interpreted as a single-cause mechanism. It instead represents a convergent cellular response to genetic vulnerability, endocrine disruption, oxidative stress, inflammation, epigenetic instability, and microenvironmental failure.

The clinical meaning of apoptosis depends on etiology and reversibility. In some patients, apoptosis may be an appropriate quality-control process that removes genetically or meiotically defective germ cells; in others, it may reflect potentially modifiable stress-induced depletion. This distinction is central to biomarker development and to the safe translation of anti-apoptotic therapies.

Future progress will require validated biomarker panels, single-cell and spatial omics, and prospective multicenter studies that stratify patients by mechanism rather than by histology alone. Until such stratification is available, apoptosis-targeted therapy should be considered experimental, and clinical management should focus on identifying reversible upstream contributors while accurately counseling patients about sperm retrieval prognosis.
