# Supplementary material for: Apoptosis in non-obstructive azoospermia: pathway crosstalk, cell-type vulnerability, and translational implications
Source: Front Endocrinol (Lausanne). 2026 Jun 26;17:1683905. doi: 10.3389/fendo.2026.1683905 (PMC13350508; doi:10.3389/fendo.2026.1683905)
Supplement: Supplementary file 1 [file Table1.docx]

**Supplementary Table 1. Abbreviations Used in the Review “Apoptosis in Non-Obstructive Azoospermia: Pathway Crosstalk, Cell-Type Vulnerability, and Translational Implications”**

**Abbreviation Full Term (English)**

| **AAV** | **Adeno-Associated Virus** |
| --- | --- |
| **Apaf-1** | **Apoptotic Protease-Activating Factor 1** |
| **AZF** | **Azoospermia Factor (AZFa/b/c)** |
| **BAX** | **BCL2-Associated X Protein** |
| **BCL2** | **B-cell lymphoma 2** |
| **BTB** | **Blood–Testis Barrier** |
| **cfDNA** | **Cell-Free DNA** |
| **circRNA** | **Circular RNA** |
| **DISC** | **Death-Inducing Signaling Complex** |
| **ER** | **Endoplasmic Reticulum** |
| **FADD** | **Fas-Associated Death Domain** |
| **FasL** | **Fas Ligand** |
| **FSH** | **Follicle-Stimulating Hormone** |
| **HPG axis** | **Hypothalamic–Pituitary–Gonadal Axis** |
| **HS** | **Hypospermatogenesis** |
| **IAP** | **Inhibitor of Apoptosis Protein** |
| **ICSI** | **Intracytoplasmic Sperm Injection** |
| **LH** | **Luteinizing Hormone** |
| **lncRNA** | **Long Noncoding RNA** |
| **MA** | **Maturation Arrest** |
| **micro-TESE** | **Microdissection Testicular Sperm Extraction** |
| **miRNA** | **MicroRNA** |
| **MOMP** | **Mitochondrial Outer Membrane Permeabilization** |
| **NOA** | **Non-Obstructive Azoospermia** |
| **OA** | **Obstructive Azoospermia** |
| **piRNA** | **PIWI-Interacting RNA** |
| **ROS** | **Reactive Oxygen Species** |
| **SCOS** | **Sertoli Cell-Only Syndrome** |
| **scRNA-seq** | **Single-Cell RNA Sequencing** |
| **Smac/DIABLO** | **Second Mitochondria-Derived Activator of Caspases / DIABLO** |
| **SSC** | **Spermatogonial Stem Cell** |
| **tBid** | **Truncated Bid** |
| **TNFR1** | **Tumor Necrosis Factor Receptor 1** |
| **UPR** | **Unfolded Protein Response** |
| **XIAP** | **X-linked Inhibitor of Apoptosis Protein** |
| **YCM** | **Y-Chromosome Microdeletion** |
